# Age‐associated adipose tissue inflammation promotes monocyte chemotaxis and enhances atherosclerosis

**DOI:** 10.1111/acel.13783

**Published:** 2023-01-23

**Authors:** Jianrui Song, Diana Farris, Paola Ariza, Smriti Moorjani, Mita Varghese, Muriel Blin, Judy Chen, Daniel Tyrrell, Min Zhang, Kanakadurga Singer, Morgan Salmon, Daniel R. Goldstein

**Affiliations:** ^1^ Department of Internal Medicine, Division of Cardiovascular Medicine University of Michigan Ann Arbor Michigan USA; ^2^ Department of Pediatrics, Division of Endocrinology University of Michigan Ann Arbor Michigan USA; ^3^ Graduate Program in Immunology University of Michigan Ann Arbor Michigan USA; ^4^ Department of Biostatistics University of Michigan Ann Arbor Michigan USA; ^5^ Department of Cardiac Surgery University of Michigan Ann Arbor Michigan USA; ^6^ Department of Microbiology and Immunology University of Michigan Ann Arbor Michigan USA

**Keywords:** atherosclerosis, inflammation, macrophage, monocyte, visceral adipose tissue

## Abstract

Although aging enhances atherosclerosis, we do not know if this occurs via alterations in circulating immune cells, lipid metabolism, vasculature, or adipose tissue. Here, we examined whether aging exerts a direct pro‐atherogenic effect on adipose tissue in mice. After demonstrating that aging augmented the inflammatory profile of visceral but not subcutaneous adipose tissue, we transplanted visceral fat from young or aged mice onto the right carotid artery of *Ldlr* ^−/−^ recipients. Aged fat transplants not only increased atherosclerotic plaque size with increased macrophage numbers in the adjacent carotid artery, but also in distal vascular territories, indicating that aging of the adipose tissue enhances atherosclerosis via secreted factors. By depleting macrophages from the visceral fat, we identified that adipose tissue macrophages are major contributors of the secreted factors. To identify these inflammatory factors, we found that aged fat transplants secreted increased levels of the inflammatory mediators TNFα, CXCL2, and CCL2, which synergized to promote monocyte chemotaxis. Importantly, the combined blockade of these inflammatory mediators impeded the ability of aged fat transplants to enhance atherosclerosis. In conclusion, our study reveals that aging enhances atherosclerosis via increased inflammation of visceral fat. Our study suggests that future therapies targeting the visceral fat may reduce atherosclerosis disease burden in the expanding older population.

## Graphical abstract

Aging increases visceral fat inflammation including the increase of adipose tissue macrophages (MΦ). The macrophages in aged visceral fat then secrete increased inflammatory mediators specifically TNFα, CCL2 and CXCL2, which consequently promote an atherogenic phenotype on circulating monocytes. The monocytes gain increased expression of inflammatory markers, chemokine receptors and migration related genes, which leads to enhanced migration of monocytes towards the artery wall to promote atherogenesis.

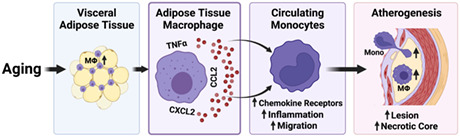

## 1 INTRODUCTION

Aging exerts multiple effects to disrupt organ homeostasis to enhance diseases (Barbe‐Tuana et al., 2020; Ungvari et al., 2018; Zhang et al., 2020). A key disease associated with aging is atherosclerosis, although how biological aging impacts atherogenesis is not fully understood (Tyrrell & Goldstein, 2021; Wang & Bennett, 2012). Aging may promote atherogenesis by altering several interacting factors such as impaired TLR function in circulating immune cells (Shaw et al., 2013), reductions in lipolysis (Camell et al., 2017), impaired mitochondrial function within the vasculature (Tyrrell et al., 2020; Vendrov et al., 2017; Yu et al., 2017), and via increasing the inflammatory profile of adipose tissue (Lumeng et al., 2011). Investigating the complex mechanisms by which aging could impact atherosclerosis is difficult due to the compounding influence of cardiovascular risk factors such as hyperlipidemia and hypertension that increase in prevalence with aging. However, there is emerging evidence indicating that biological aging, independent of chronic exposure to hyperlipidemia, enhances atherogenesis (Tyrrell et al., 2020). Given the likely multiple interactions between all the potential age‐related atherogenic promoting factors, the contribution of each age‐related factor to atherosclerosis remains unclear.

Aging induces multiple changes in adipose tissue including increased fat mass, adipocyte size, macrophage infiltration, and an increased production of inflammatory cytokines and chemokines (Adlung et al., 2019; Flegal et al., 2012; Lumeng et al., 2011). Increased adiposity has been associated with metabolic dysfunction and subsequent atherosclerosis (McLaughlin et al., 2017; Satish et al., 2019). Furthermore, metabolic signals within adipose tissue become dysregulated with aging, specifically manifest as increased leptin and reduced adiponectin secretion (Frasca & Blomberg, 2020). This is accompanied by a reduction in lipolysis with aging, a failure to clear free fatty acids, which may activate inflammatory pathways such as NLRP3 inflammasome to promote adipose tissue inflammation (Camell et al., 2017; Jaitin et al., 2019; Lee & Dixit, 2020; Youm et al., 2013). The increased inflammatory profile of adipose tissue with aging likely contributes to systemic inflammation, insulin resistance, and metabolic dysfunction. Clearly, aging alters the immune metabolic profile of adipose tissue, which has been proposed to enhance several age‐associated diseases (Lee & Dixit, 2020). Yet, whether these age‐related immune metabolic changes within adipose tissue directly impact atherogenesis remains unclear. This lack of clarity stems from the fact that experiments have not been conducted to isolate specific age‐related factors and examine their role in promoting atherogenesis. Clarification of the contribution of each age‐specific factor in enhancing atherosclerosis will provide fundamental information that will assist in age‐specific therapies to reduce atherosclerosis in the growing population of older individuals.

Here, we investigated the role of adipose tissue in age‐enhanced atherosclerosis. Specifically, we employed a fat transplant model in mice to examine the direct effect of aging on the visceral fat depot in enhancing atherosclerosis. We focused on the visceral fat as we found that this depot becomes more inflamed than the subcutaneous depot with aging. Our results show that, with aging, visceral fat secretes inflammatory factors that enhance atherosclerosis in both a paracrine and endocrine manner. Our study identifies that macrophages within the visceral fat with aging are the major producers of inflammatory mediators. Finally, our study reveals that the combined inhibition of the inflammatory mediators TNFα, CXCL2, and CCL2 impedes the ability of aged visceral fat transplants to enhance atherogenesis, which indicates that the aged visceral fat promotes atherogenesis predominantly by secreting TNFα, CXCL2, and CCL2.

## 2 RESULTS

### 2.1 Visceral fat depot exhibits an augmented inflammatory profile with aging

**FIGURE 1:**
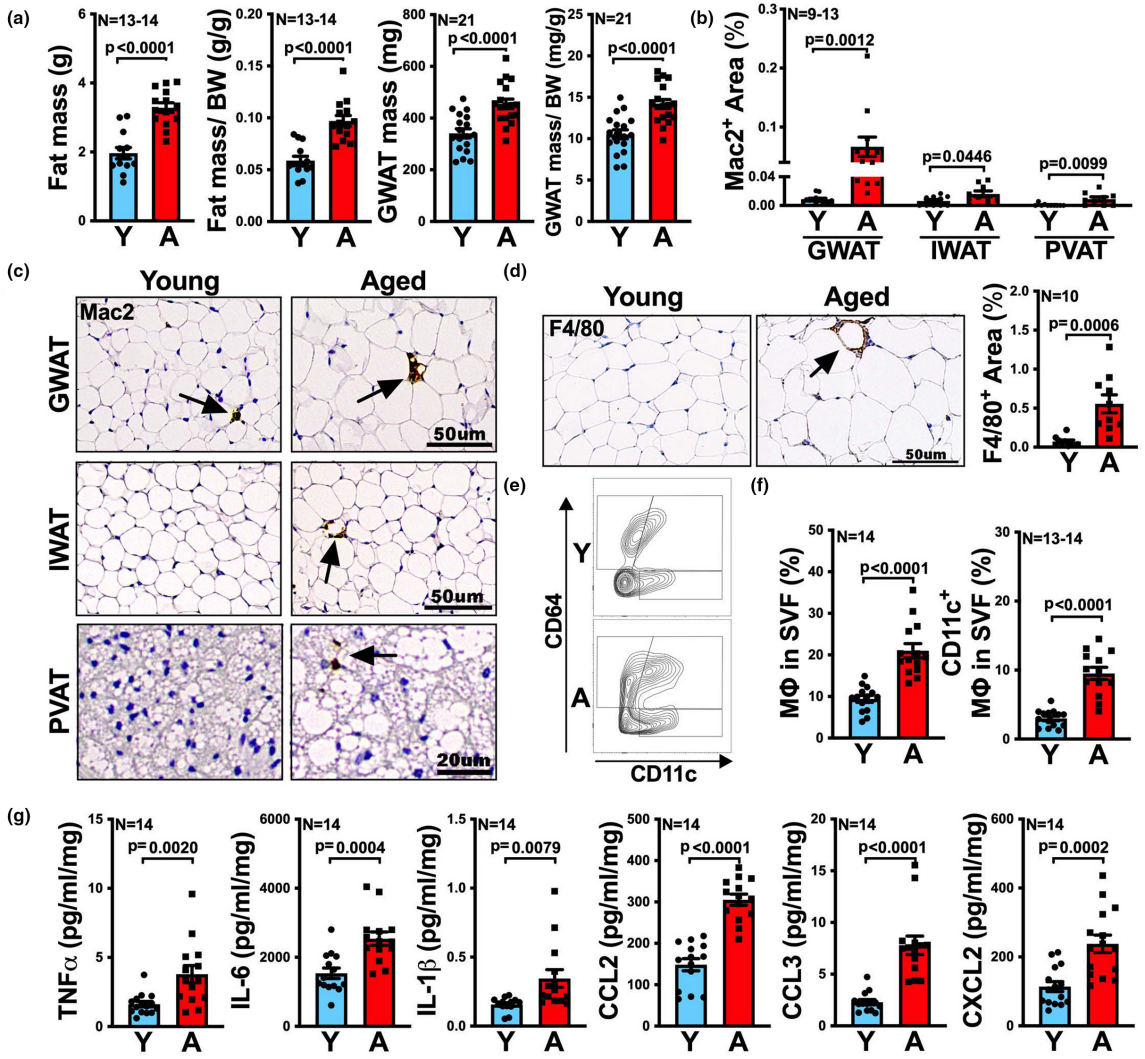
With aging, viseral fat exhibits increased macrophage numbers and secretes more inflammatory cytokines and chemokines. (a) The total body fat of young (Y) and aged (A) mice was measured by body composition analyzer, EchoMRI. The gonadal white adipose tissue (GWAT) was collected and weighed after euthanizing the mice. The fat mass (*n* = 13‐14/group) and GWAT mass (*n* = 21/group) to body weight (BW) ratio were calculated. (b, c) Representative images of Mac2‐stained visceral (GWAT), subcutaneous (inguinal white adipose tissue, IWAT) and perivascular adipose tissue (PVAT) sections of Y and A mice are shown (c), and the percentage of Mac2 positive areas was quantified and is shown in (b), *n* = 9–13/group. (d) Representative images of F4/80‐stained visceral fat (GWAT) sections of young and aged mice. Quantification of the percentage of F4/80 positive areas is shown on the right (*n* = 10/group). (e, f) Flow cytometric analysis of total adipose tissue macrophage (MΦ) and CD11c^+^ adipose tissue macrophages in visceral fat (GWAT) of Y and A mice. Representative FACS contour plots of CD64^+^ and CD64^+^CD11c^+^ adipose tissue macrophages are shown in (e), and the quantification of total (CD64^+^) and CD11c^+^ adipose tissue macrophages (CD64^+^CD11c^+^) in stromal vascular fraction (SVF) is shown in (f), *n* = 13‐14/group. (g) Protein levels of TNFα, IL‐6, IL‐1β, CCL2, CCL3, and CXCL2 in the tissue culture medium of visceral fat (GWAT) from Y and A mice were determined by multiplex assay (*n* = 14/group). Results are presented as means ± SEM. Unpaired two‐tailed Student's *t*‐test was used for statistical analysis.

We hypothesized that aging impacts adipose tissue via increased inflammation to promote atherosclerosis. Hence, we first compared fat depots of young (3–4 months) and aged (19–21 months) C57BL/6 male mice. With aging, there was an increase in total fat mass and visceral fat mass (i.e., gonadal white adipose tissue, GWAT) shown in Figure 1a. Given that adipose tissue inflammation correlates with adipose tissue macrophages (Russo & Lumeng, 2018; Zeyda & Stulnig, 2007), we enumerated macrophages in visceral (GWAT was used as visceral fat in this study), subcutaneous (i.e., inguinal white adipose tissue, IWAT), and perivascular adipose tissue (PVAT) between young and aged mice, via immunohistochemistry staining of Mac2, a widely used macrophage marker (Leenen et al., 1994; Rong et al., 2003). In all three sites, Mac2 staining increased with aging (Figure 1b,c), suggesting that adipose tissue macrophage (defined as Mac2^+^ cells) numbers increase with age. We also noted that the aged visceral fat depot exhibited the highest level of Mac2 staining, compared to the other two aged fat depots (Figure 1b,c).

Our findings were not restricted to the C57BL/6 genetic background or the male sex, as visceral fat from UM‐HET3 (Miller et al., 2007), four‐way crossed outbred, aged male mice or aged female C57BL/6 mice also exhibited increased adipose tissue macrophage numbers compared to the young ones, shown by Mac2 staining (Figure S1a,b).

To confirm that aging increases macrophage expansion in visceral fat, in addition to Mac2 staining, we also performed immunohistochemical staining for F4/80, as well as flow cytometry for CD64, which are both alternative macrophage markers. Consistently, aged visceral fat showed a significant (*p* = 0.0006) >fivefold increased expression of F4/80 (Figure 1d), as well as a significant (*p* < 0.0001) increase of CD64^+^ cells in stromal vascular fraction (SVF) (Figure 1e,f), compared to young control. Moreover, the proportion of infiltrated inflammatory adipose tissue macrophage (CD64^+^CD11c^+^; Drutman et al., 2012; Lumeng et al., 2007; Singer et al., 2014, 2015) was also found significantly increased (twofold to threefold, *p* < 0.0001) in aged visceral fat SVF compared to that from young mice (Figure 1e,f).

Finally, as visceral and subcutaneous fat depots are the two main white adipose tissue depots that contribute to metabolic health (Luong et al., 2019), we examined how aging modulates the secretion of inflammatory mediators in those two fat depots. Specifically, we measured the proteins levels in culture media of visceral and subcutaneous fat. In subcutaneous fat, we found no significant difference in pro‐inflammatory cytokines and chemokines secretion with aging (Figure S1d). However, in visceral fat, aging led to a significant (approximate twofold to threefold) increase in the secretion of TNFα, IL‐6, IL‐1β, CCL2, CCL3, and CXCL2 (Figure 1g). Increase was also shown in the secretion of leptin in visceral but not subcutaneous fat (Figure S1e). Of note, we found that visceral fat from aged female mice also exhibited increased secretion of inflammatory mediators, recapitulating our findings in male mice (Figure S1c,e).

Taken together, the results above indicate that aging increases macrophage numbers and the secretion of inflammatory mediators within the visceral fat, implying that aging increases the inflammatory profile of visceral fat depot.

### 2.2 Aged visceral adipose tissue enhances atherosclerotic plaque formation in local and distal vascular territories

**FIGURE 2:**
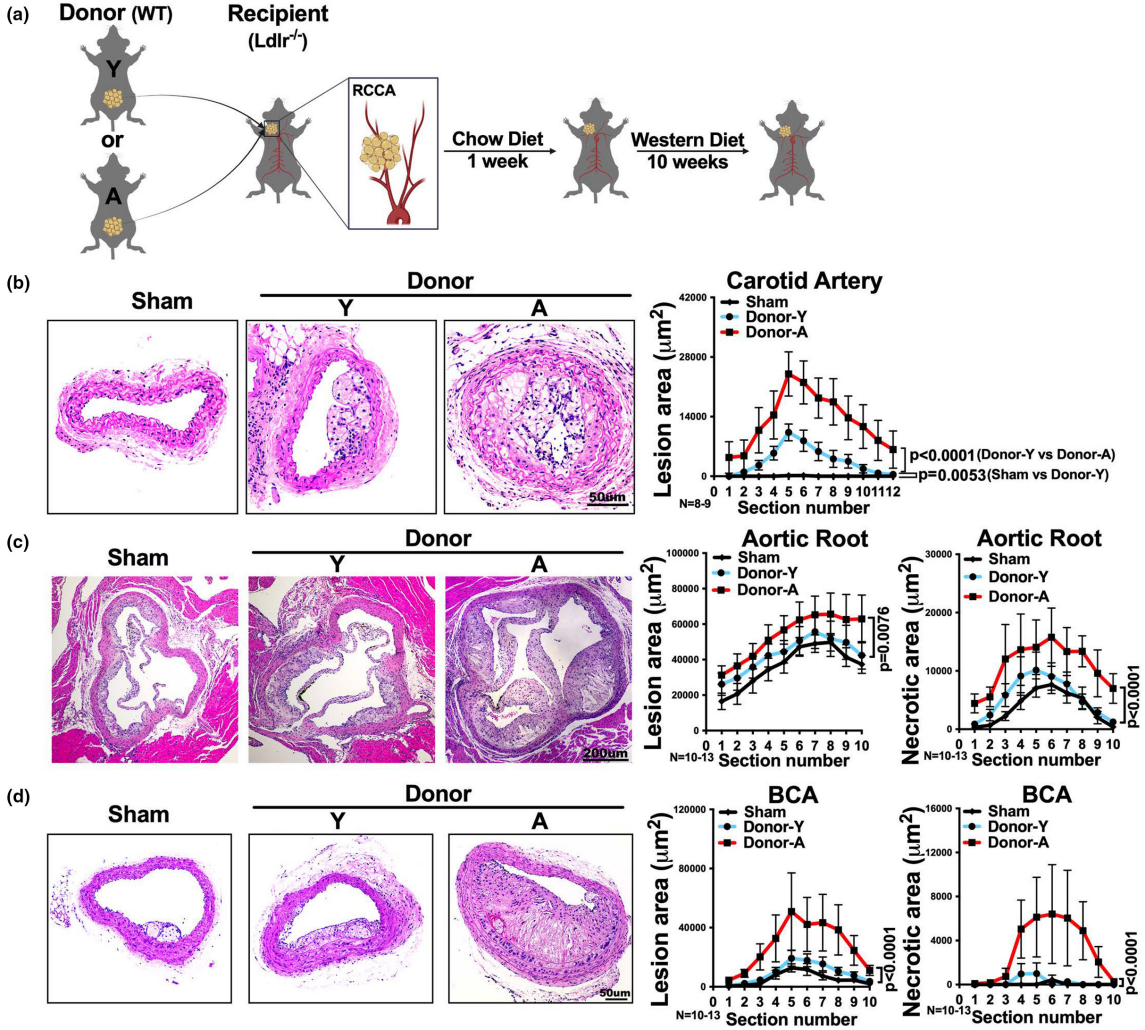
Visceral fat from aged donor mice increases recipient atherosclerotic lesion size and necrotic core size after transplantation. (a) Schematic for fat transplant model: visceral fat from young (Y) or aged (A) WT donor mice was transplanted onto the right common carotid artery (RCCA) of young *Ldlr* ^−/−^ recipient mice. The recipients were then fed a low fat chow diet for a week followed by a western diet for 10 weeks. (b–d) Representative images of H&E stained (b) right carotid artery, (c) aortic root and (d) brachiocephalic artery (BCA) sections of *Ldlr* ^−/−^ mice that received sham surgery or visceral fat transplants from young (Donor‐Y) or aged (Donor‐A) donor mice. The quantifications of lesion area (b–d) and necrotic area (c, d) in each section are shown on the right (*n* = 8‐13/group). Results are presented as means ± SEM. Two‐way ANOVA followed by Tukey's post hoc test was used for statistical analysis. *p* values indicate the main effect of the comparison between Donor‐Y vs. Donor‐A unless specifically indicated.

As we found that aging increased the inflammatory profile of visceral fat, we next examined if aging increases the atherogenic potential of visceral fat by employing a fat transplant model (Ohman et al., 2011), as shown in Figure 2a. In this model, fat is transplanted onto the right carotid artery of recipient mice. We chose this model since it is not clear if the aging of visceral fat would lead to the secretion of factors that act locally on adjacent vasculature, or systemically on distal vascular sites. Also, as far as we know, this is the only available model to directly examine the atherogenic potential of aging on visceral fat depot, independent from aging's effects on circulating immune cells, vasculature, and systemic lipid metabolism. Hence, we transplanted 100 mg of visceral fat from young or aged donor mice onto the right carotid artery of young *Ldlr* ^−/−^ recipient mice, an established murine model of atherosclerosis (Daugherty et al., 2017). We also included a group of young *Ldlr* ^−/−^ mice that received a sham operation. After transplantation, the recipient mice were given western diet for 10 weeks to increase circulating lipid levels and enhance atherosclerosis (Daugherty et al., 2017). At the end, recipient mice were euthanized, and the degree of atherosclerosis was assessed in the right carotid artery and other vascular beds including aortic root and brachiocephalic artery (BCA). Note that, during the western diet feeding period, fasting cholesterol, triglyceride, and insulin levels and the gain in body weight were similar between recipients that received sham surgery or fat transplants from young or aged donors (Figure S2).

We found that recipients that received a visceral fat transplant from aged donors, exhibited more than threefold increase in atherosclerotic lesion size in the right carotid artery that was in direct contact with the fat transplant, compared to recipients that received young fat transplants (Figure 2b). Young fat transplants still enhanced atherosclerosis in the right carotid artery, compared to sham which did not result in any local atherosclerotic lesions (Figure 2b). Interestingly, recipients with aged fat transplants also exhibited a significant approximate twofold increase in atherosclerotic lesion size distally in both aortic root (Figure 2c) and BCA (Figure 2d), compared to recipients that received young fat transplants. Additionally, in both aortic root and BCA, recipients with aged fat transplants exhibited a significant (*p* < 0.0001) increase in atherosclerotic necrotic core size (more than twofold in aortic root; more than 13‐fold in BCA), a surrogate of plaque instability (Fernandez‐Hernando et al., 2007; Moore et al., 2013), compared to recipients with young fat transplants (Figure 2c,d). Visceral fat transplantation from young donors to recipient mice did not cause a significant increase in either atherosclerotic lesion size or necrotic core size, in either aortic root or BCA, compared to sham recipients (Figure 2c,d). This suggests that the fat transplant technique itself induces a local, but not systemic, effect to promote atherosclerosis.

Taken together, these results indicate that with aging, the visceral adipose tissue enhances atherogenesis in both local (i.e., carotid artery) and distal (i.e., aortic root and BCA) vascular territories.

### 2.3 Aging of the visceral fat promotes macrophage infiltration into adjacent and distal atherosclerotic lesions

**FIGURE 3:**
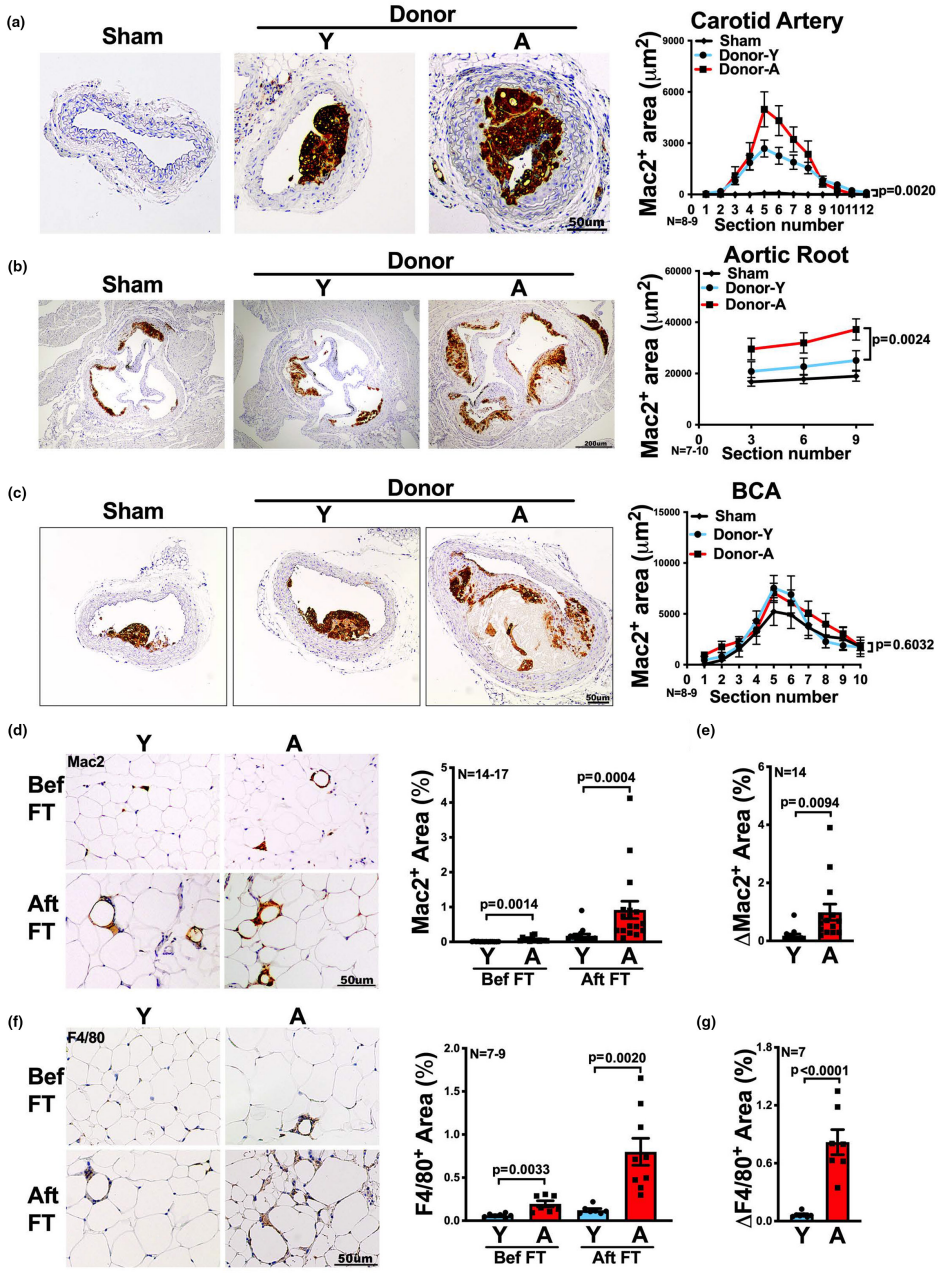
Visceral fat transplants from aged mice increase macrophage numbers within athersclerotic lesions and fat transplants after transplantation. (a–c) Representative images of Mac2‐stained (a) right carotid artery, (b) aortic root and (c) brachiocephalic artery (BCA) sections of *Ldlr* ^−/−^ mice that received sham surgery or visceral fat transplants from young (Donor‐Y) or aged (Donor‐A) mice. The quantification of Mac2 positive area in each section is shown on the right (*n* = 7‐10/group). (d, f) Representative images of (d) Mac2 or (f) F4/80 stained visceral fat (GWAT) sections of young (Y) or aged (A) donors, before (bef) and after (aft) fat transplant (FT) surgery, are shown. The quantification of Mac2 (*n* = 14‐17/group) or F4/80 (*n* = 7‐9/group) positive area is shown on the right. (e, g) The change in the percentage of (e) Mac2 or (g) F4/80 positive area after FT, compared to before FT, in young (Y) or aged (A) visceral fat is shown (*n* = 7 or 14 / group). Results are presented as means ± SEM. Two‐way ANOVA followed by Tukey's post hoc test was used for statistical analysis in (a–c). *p* values indicate the main effect between Donor‐Y vs. Donor‐A groups. Unpaired two‐tailed Student's *t*‐test was used for statistical analysis in (d–g).

Macrophages are instrumental for atherogenesis (Moore et al., 2013), and their content within atherosclerotic plaques are a surrogate of plaque instability (Fernandez‐Hernando et al., 2007; Moore et al., 2013). Hence, we measured macrophage proportions within the atherosclerotic lesions. Recipient mice with aged fat transplants exhibited a significant increase in macrophage staining, shown as total area of Mac2^+^ staining, in both right carotid artery and aortic root, though not BCA, compared to recipients with young fat transplants (Figure 3a–c). When we calculated the percentage of Mac2^+^ area, recipients with aged fat transplants showed a significant increase in aorta root (*p* = 0.0008) and BCA (*p* = 0.0055), though not in carotid artery, compared to recipients with young fat transplants (Figure S3). A potential explanation for the lack of difference in total Mac2^+^ area in BCA lesions between recipients with young and recipients with aged fat transplants is that, the lesions in recipients with aged fat transplants exhibited much larger necrotic area than recipients with young fat transplants (Figure 2d).

Prior to transplantation, we noted that macrophage staining in visceral fat increased threefold to sevenfold in aged mice compared to young mice (Figure 3d,f), shown by macrophage markers Mac2 and F4/80. By the end of the experiment, fat transplants were collected and stained again for those two macrophage markers. Fat transplants from aged donors still exhibited increased macrophage staining compared to young fat transplants, with a fivefold to sevenfold increase (Figure 3d,f). Interestingly, fat transplants from aged mice exhibited a 4–13‐fold increase in macrophage staining compared to the pre‐transplant state, which was significantly higher than the change from baseline in young fat transplants (*p* = 0.0094 for Mac‐2, *p* < 0.0001 for F4/80, Figure 3e,g).

Overall, with aging, visceral fat enhances macrophage infiltration into local and distal atherosclerotic lesions.

### 2.4 Macrophages are key contributors to the inflammatory profile of visceral fat with aging

**FIGURE 4:**
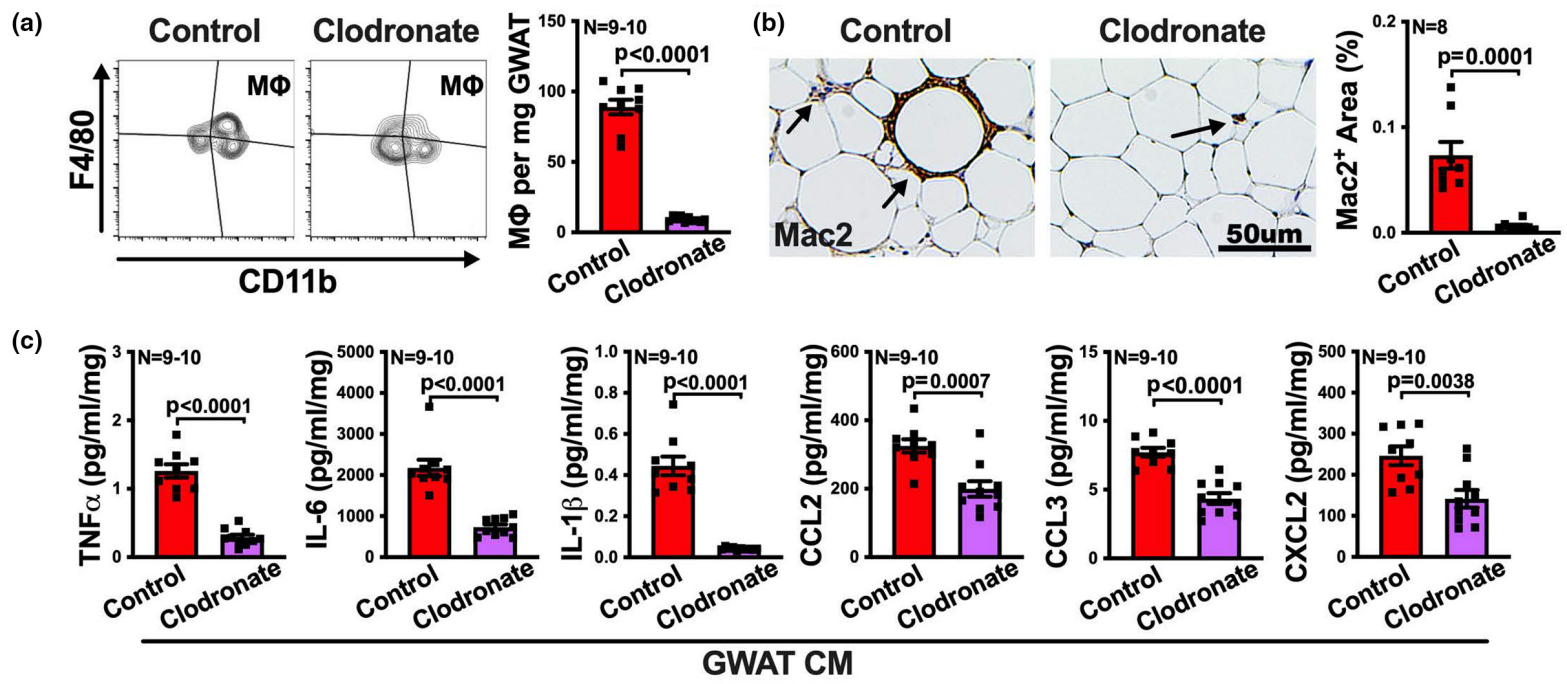
Macrophages are major contributors to the inflammatory profile in visceral adipose tissue with aging. Aged mice were treated with control liposome or liposome‐encapsulated clodronate by intraperitoneal injection, and then the macrophage depletion efficiency in visceral fat (GWAT) was determined, as well as the secretion of inflammatory cytokines and chemokines. (a) Representative FACS contour plots of macrophages (F4/80^+^CD11b^+^) in visceral fat, and the numbers of macrophages per milligram tissue are shown (*n* = 9‐10/group). (b) Representative images of Mac2‐stained visceral fat sections are shown, and the percentage of Mac2 positive areas was quantified and is shown on the right (*n* = 8/group). (c) Levels of TNFα, IL‐6, IL‐1β, CCL2, CCL3, and CXCL2 in the tissue culture medium (CM) of visceral fat were determined by multiplex assay (*n* = 9‐10/group). Results are presented as means ± SEM. Unpaired two‐tailed Student's *t*‐test was used for statistical analysis.

Given the above results, we hypothesized that macrophage is the key cell type responsible for the enhanced inflammatory profile of visceral fat with aging. We tested this hypothesis by depleting macrophages from the visceral fat of aged mice via administering clodronate‐encapsulated liposomes to aged mice. Empty liposomes were given to control aged mice. First, we confirmed macrophage depletion in the visceral fat by flow cytometry and immunohistochemistry staining (Figure 4a,b). Next, we cultured visceral fat from clodronate treated or control aged mice and measured the protein levels of inflammatory mediators in culture media. Macrophage‐depleted visceral fat exhibited a 2–10‐fold reduction in the secretion of all measured inflammatory mediators including TNFα, IL‐6, IL‐1β, CCL2, CCL3, and CXCL2, compared to the control (Figure 4c). These results indicate that macrophage is the major cell type that contributes to the inflammatory profile of aged visceral fat.

### 2.5 Senescence is involved in the enhanced inflammatory profile of visceral fat with aging

Aging may lead to increased cellular senescence within fat depots (Tchkonia et al., 2010). Senescence causes increased secretion of inflammatory mediators known as the senescence associated secretory phenotype (SASP; Tchkonia et al., 2013). The enhanced secretion of inflammatory mediators by visceral fat from aged mice (Figure 1g) is compatible with SASP. Therefore, we determined senescence in young and aged visceral fat by measuring the senescence‐associated β‐galactosidase activity (Debacq‐Chainiaux et al., 2009). Visceral fat from aged mice showed significantly increased β‐galactosidase activity (*p* < 0.0001, Figure S4a,b). To assess whether decreasing senescence would reduce the inflammatory profile of visceral fat with aging, we administrated senolytics (Kirkland & Tchkonia, 2017) dasatinib and quercetin (known as D + Q), an established senolytic protocol (Palmer et al., 2019; Xu et al., 2018), in aged mice. By measuring the inflammatory mediators in visceral fat tissue lysates of D + Q or vehicle control treated aged mice, we found that D + Q caused a 1.5–5.5‐fold reduction in all the measured inflammatory mediators (Figure S4c), which suggests that senescence is involved in enhancing the inflammatory profile of aged visceral fat.

### 2.6 Adipose tissue macrophages are the major contributors in aged fat transplants to enhance atherosclerosis

**FIGURE 5:**
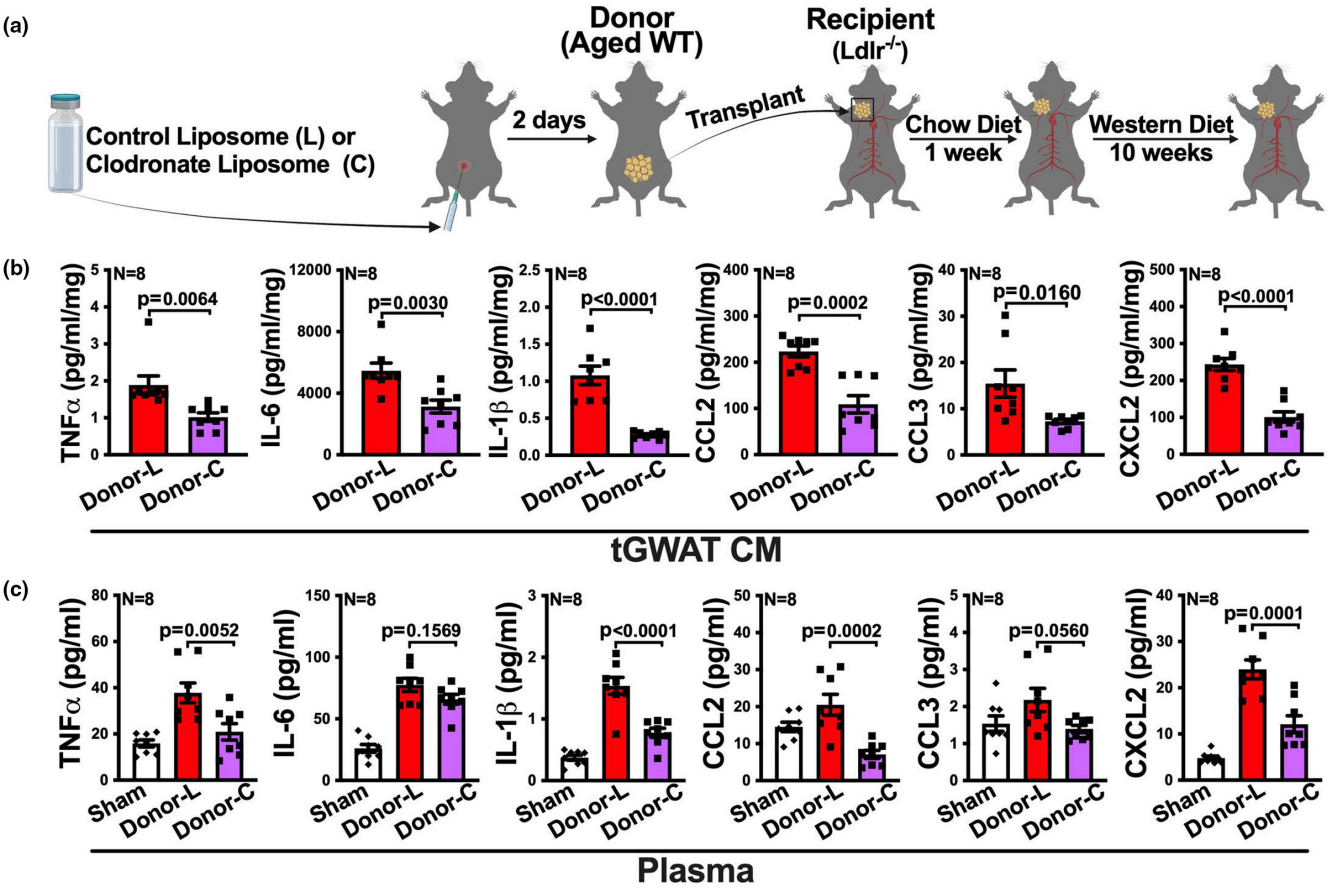
Macrophages within the aged fat transplant contribute to the inflammatory profile of the fat transplant and systemically within the recipient. (a) Experimental schematic: aged WT donor mice were treated with control liposome (L) or liposome‐encapsulated clodronate (C) through intraperitoneal injection. 2 days later, visceral fat (GWAT) transplants were collected from the donor mice and transplanted onto the RCCA of young *Ldlr* ^−/−^ recipient mice, followed by a low fat chow diet for a week and then a western diet for 10 weeks. (b, c) The transplanted visceral fat (tGWAT) from the recipients and plasma from sham mice and the recipients were collected at the end of the experiment, and levels of TNFα, IL‐6, IL‐1β, CCL2, CCL3, and CXCL2 in (b) tissue culture medium (CM) of tGWAT and (c) plasma were determined by multiplex assay (*n* = 8/group). Results are presented as means ± SEM. Unpaired two‐tailed Student's *t*‐test was used in (b) and one‐way ANOVA followed by Tukey's post hoc test was used in (c) for statistical analysis.

After confirming that macrophages are the key contributors of the inflammatory profile of aged visceral fat, we investigated whether macrophages are importantly involved in atherogenesis that was enhanced by aged visceral fat. Clodronate or control liposome treated aged visceral fat was transplanted to *Ldlr* ^−/−^ recipients (Figure 5a). By the end of the experiment, fat transplants were collected and cultured, and then we measured the protein levels of inflammatory mediators in the culture media. The culture media of clodronate‐treated fat transplants showed a twofold to fivefold decrease in the secretion of all measured inflammatory mediators (Figure 5b), compared to culture media of control liposome‐treated fat transplants. Additionally, recipients of clodronate treated aged fat transplants exhibited a significant, approximate twofold to threefold reduction in plasma levels of most measured inflammatory cytokines and chemokines TNFα, IL‐1β, CCL2, and CXCL2, except IL‐6 and CCL3, compared to recipients with control liposome‐treated fat transplants (Figure 5c).

**FIGURE 6:**
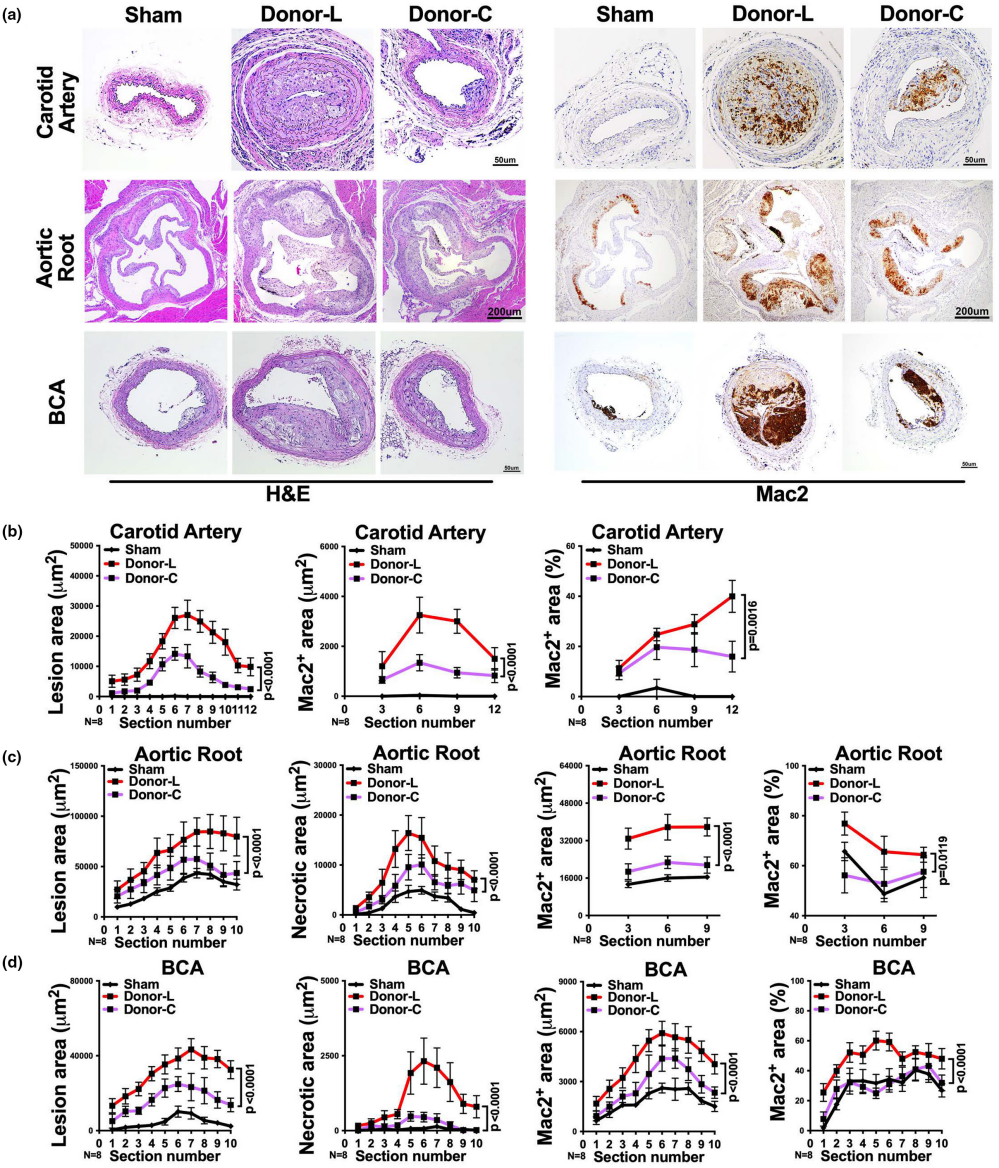
Macrophages within the aged fat transplant contribute to atherogenesis. Aged WT donor mice were treated with clodronate liposomaes according to the experimental schema described in Figure 5a. (a) Representative images of H&E or Mac2 stained right carotid artery, aortic root and BCA sections of *Ldlr* ^−/−^ mice that received sham surgery or visceral fat transplants from control liposome treated (Donor‐L) or clodronate‐liposome treated (Donor‐C) aged donor mice. (b–d) The quantifications of lesion area, necrotic area, Mac2 positive area, and the percentage of Mac2 positive area in each section of (b) right carotid artery, (c) aortic root, and (d) BCA are shown (*n* = 8/group). *Note*: no necrosis was observed in carotid artery. Results are presented as means ± SEM. Two‐way ANOVA followed by Tukey's post hoc test was used for statistical analysis. *p* values indicate the main effect between groups of Donor‐L and Donor‐C.

Importantly, recipients with clodronate treated fat transplants exhibited a significant decrease in atherosclerotic lesions and necrotic area (*p* < 0.0001), compared to control recipients, in carotid artery, aortic root, and BCA (Figure 6a–d). Note, no necrosis was observed in lesions of carotid artery. In addition, recipients with clodronate treated aged fat transplants exhibited a ~twofold reduction in macrophage staining in all three vascular territories, compared to control recipients (Figure 6a–d).

Taken together, these results suggest that adipose tissue macrophages substantially contribute to visceral fat‐enhanced atherogenesis with aging.

### 2.7 Aged visceral fat transplant promotes monocyte chemotaxis predominantly by secreting TNFα, CXCL2 and CCL2

As we noted that recipients with aged fat transplants showed increased macrophages in both fat transplants and atherosclerotic lesions (Figure 3), we reasoned that aged fat transplant increases monocyte infiltration into tissues, including infiltration into the fat transplants and atherosclerotic lesions. To test this hypothesis, we performed chemotaxis assay, with monocytes that were isolated from the bone marrow of young C57BL/6 mice, to compare the monocyte chemotaxis‐inducing potential of the conditioned media obtained after ex vivo culturing the young or aged fat transplants.

**FIGURE 7:**
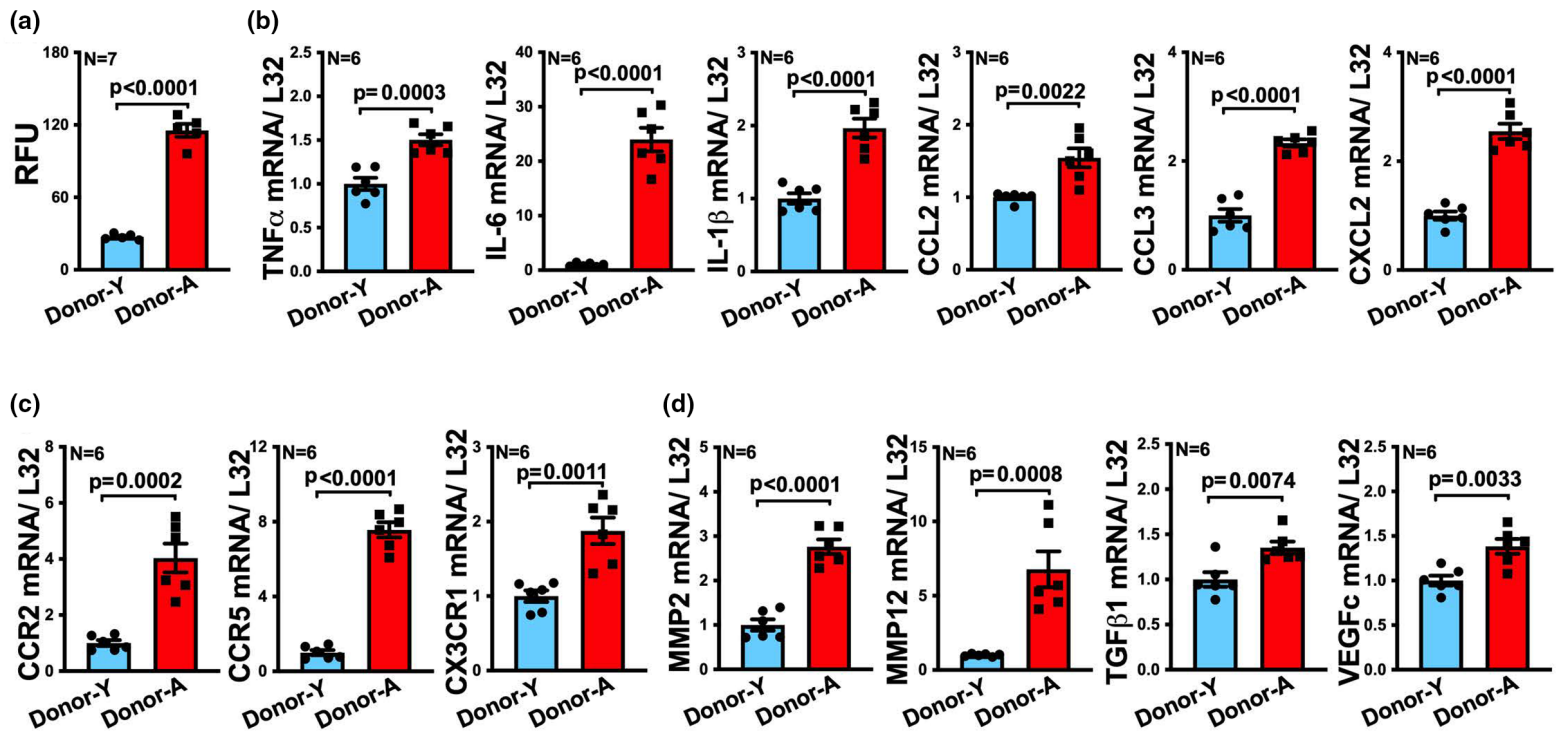
Tissue culture media of aged visceral fat transplants enhance monocyte chemotaxis and monocyte expression of inflammatory and migratory genes. (a) The ability of monocytes to migrate toward the tissue culture media of explanted young (Donor‐Y) or aged (Donor‐A) visceral fat transplants (tGWAT) was determined via chemotaxis assay (*n* = 7/group). RFU = relative fluorescence. (b–d) Migrated monocytes were collected after migration toward tissue culture media of young (Donor‐Y) or aged (Donor‐A) visceral fat transplants. mRNA levels of (b) inflammatory cytokines including TNFα, IL‐6, IL‐1β; and the chemokines CCL2, CCL3, and CXCL2; and (C) chemokine receptors CCR2, CCR5, and CX3CR1; and (d) genes that are known to associate with cell migration including MMP2, MMP12, TGFβ1, and VEGFc were measured by qRT‐PCR (*n* = 6 / group). Gene expression was normalized to ribosomal gene L32 (fold change shown on Y axis of plots of b–d). Results are presented as means ± SEM. Unpaired two‐tailed Student's *t*‐test was used for statistical analysis.

Culture media from aged fat transplants induced a significant >fourfold increase in monocyte chemotaxis, compared to that from young fat transplants (Figure 7a). In addition, we measured the gene expression profile of migrated monocytes that were exposed to young or aged culture media. The migrated monocytes that were exposed to culture media from aged fat transplants showed a significant 1.5–24‐fold increase in the expression of inflammatory genes including TNFα, IL‐6, IL‐1β, CCL2, CCL3, and CXCL2, compared to the monocytes exposed to young culture media (Figure 7b). We also measured the mRNA level of chemokine receptors including CCR2, CCR5, and CX3CR1, which have been implicated in monocyte transmigration and atherosclerosis (CombadièRe et al., 2008; Tacke et al., 2007), and cell migration‐related genes including MMP2, MMP12 (Chen & Parks, 2009), TGFβ1, and VEGFc (Simpson et al., 2008) in migrated monocytes. Monocytes that were exposed to aged culture media showed a 1.5–8‐fold increase in all the genes measured, compared to monocytes exposed to young culture media. These results suggest that aged fat transplant enhances monocyte migration and chemotaxis probably via the secreted mediators in the culture media.

**FIGURE 8:**
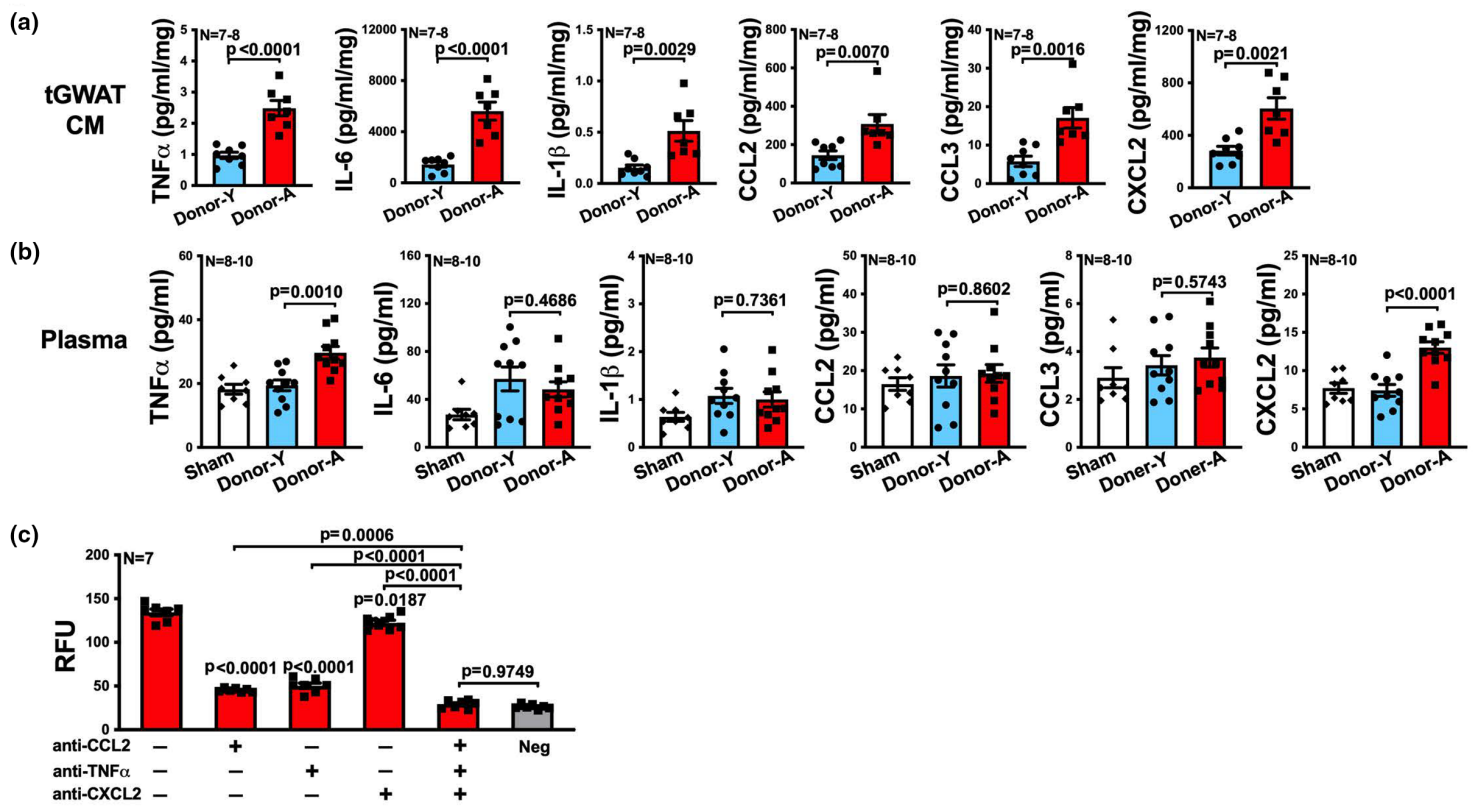
Aged visceral fat transplants induce local and systemic release of inflammatory mediators promoting monocyte chemotaxis. (a) Protein levels of cytokines, TNFα, IL‐6, IL‐1β, and chemokines CCL2, CCL3, and CXCL2 in tissue culture medium (CM) of transplanted visceral fat (tGWAT) from young (Donor‐Y) or aged (Donor‐A) mice were determined by multiplex assay (*n* = 7‐8/group). (b) Protein levels of inflammatory mediators TNFα, IL‐6, IL‐1β, CCL2, CCL3, and CXCL2 in plasma of *Ldlr* ^−/−^ mice that received sham surgery or visceral fat (GWAT) transplants from young (Donor‐Y) or aged (Donor‐A) mice were determined by multiplex assay (*n* = 8‐10/group). (c) Monoclonal antibodies against CCL2, TNFα, and/or CXCL2 or isotype control were added into the tissue culture media of aged visceral fat transplants, and then monocyte chemotaxis was determined (*n* = 7/group). Serum‐free medium was used as negative control (Neg). *p* values above histograms without brackets are *p* values in comparison to isotype control group, far left histogram. Results are presented as means ± SEM. Unpaired two‐tailed Student's *t*‐test was used in (a) and one‐way ANOVA followed by Tukey's post hoc test was used in (b, c) for statistical analysis.

To identify these mediators, we measured the cytokine/chemokine secretion profile of young and aged fat transplants in their culture media. Aged fat transplants exhibited an twofold to fourfold increase in the secretion of TNFα, IL‐6, IL‐1β, CCL2, CCL3, and CXCL2, compared to young fat transplants (Figure 8a). As aged fat transplants also promoted atherogenesis and caused increased macrophages in distal atherosclerotic lesions (Figures 2c,d and 3b,c; Figure S3), we measured the inflammatory mediators in the recipients' plasma to investigate the factors that mediate the distal effect. The plasma of recipients with aged fat transplants showed an approximate twofold increase in TNFα and CXCL2 levels, compared to recipients with young fat transplants, but no changes in the other measured factors including IL‐6, IL‐1β, CCL2, and CCL3 (Figure 8b).

Given that TNFα, a cytokine that promotes myeloid chemoattraction (Arendt et al., 2002; Murao et al., 2000), and CXCL2, a chemine that recruit myeloid cells into tissues (Kulkarni et al., 2019; Sawant et al., 2021), were increased in both the culture media of aged fat transplants and the plasma of recipients with aged fat transplants, and the well‐known importance of monocyte attracting chemokine CCL2 which also increased in culture media of aged fat transplants, we examined the role of each of these factors in monocyte chemoattraction. Specifically, we blocked TNFα, CXCL2, and CCL2 either alone or all together by adding neutralizing monoclonal antibodies to the culture media of aged fat transplants and then assessed monocyte chemotaxis relative to IgG isotype control. Either anti‐TNFα, anti‐CXCL2, or anti‐CCL2 alone significantly reduced the ability of aged culture media to induce monocyte chemotaxis, compared to IgG isotype control, although not quite to the background levels of the negative serum‐free control media (Figure 8c). However, in contrast with the twofold to threefold reduction in chemotaxis induced by either anti‐TNFα or anti‐CCL2, the reduction inducted by anti‐CXCL2 was minimal but significant (Figure 8c). Nevertheless, adding all three antibodies together resulted in a significant further decrease in monocyte migration compared to each antibody alone, and abolished the ability of aged culture media to induce monocyte chemotaxis to background levels (Figure 8c, compare samples that had all three antibodies to negative control).

In conclusion, these results indicate that aged fat transplant enhances monocyte chemotaxis predominantly by secreting TNFα, CXCL2, and CCL2.

### 2.8 Aged fat transplant promotes atherogenesis predominantly by secreting TNFα, CXCL2, and CCL2

**FIGURE 9:**
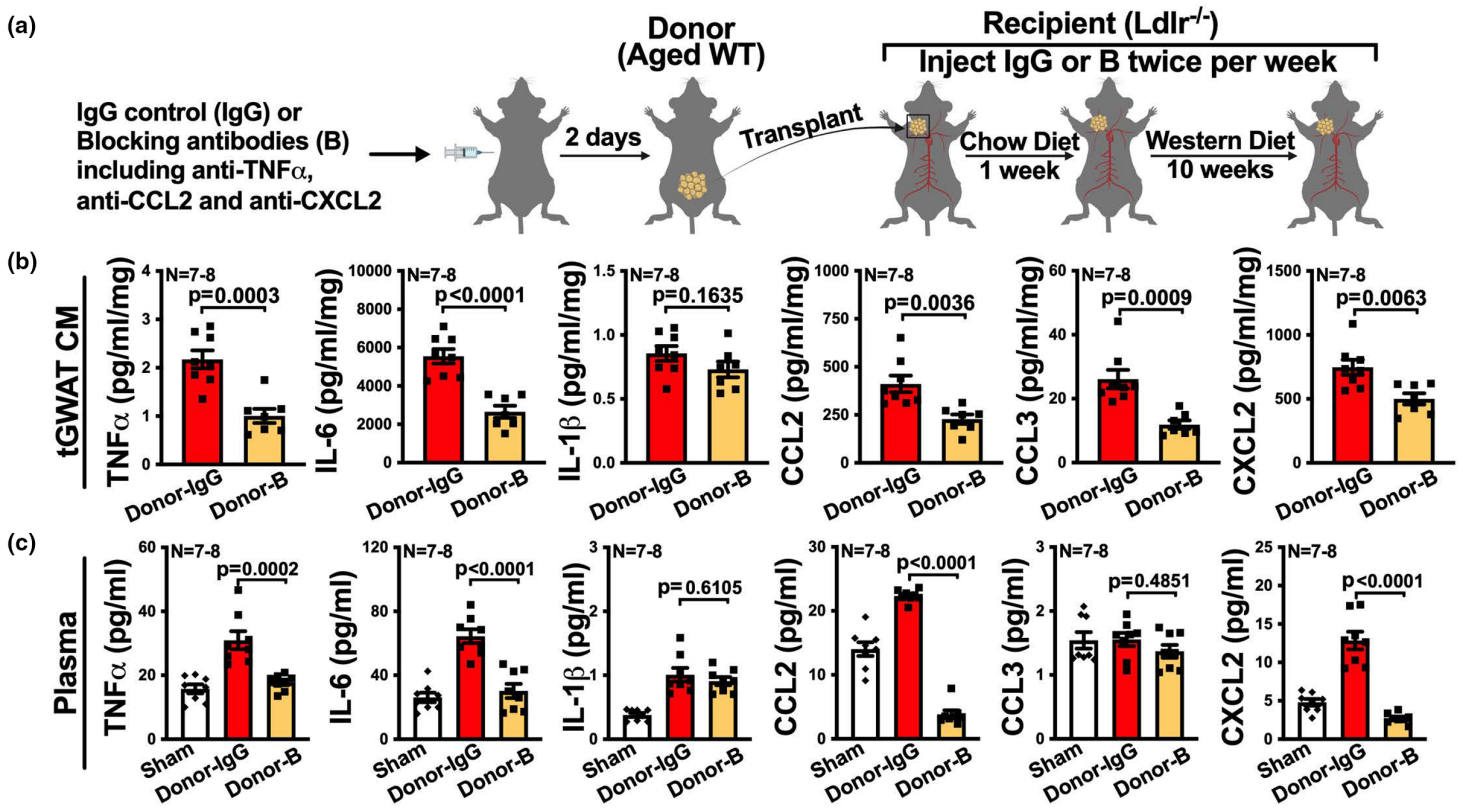
Combined blockade of TNFα, CCL2, and CXCL2 reduces the inflammatory profile of fat transplant and also reduces the systemic inflammatory profile in *Ldlr* ^−/−^ recipients. (a) Experimental schematic: aged WT donor mice were treated with IgG control (Donor‐IgG) or combined monoclonal antibodies against TNFα, CCL2, and CXCL2 (Donor‐B), 2 days later, visceral fat (GWAT) transplants were collected from the donor mice and transplanted onto the RCCA of young *Ldlr* ^−/−^ recipient mice, followed by a low fat chow diet for a week and then a western diet for 10 weeks. IgG control or TNFα, CCL2, and CXCL2 blockade were continuously injected into the recipients that received the corresponding transplants twice per week after transplantation till the end of the experiment. (b, c) The transplanted visceral fat (tGWAT) from the recipients and plasma from sham mice and the recipients were collected at the end of the experiment, and protein levels of TNFα, IL‐6, IL‐1β, CCL2, CCL3, and CXCL2 in (b) tissue culture medium (CM) of tGWAT and (c) plasma were determined by multiplex assay (*n* = 7–8). Results are presented as means ± SEM. Unpaired two‐tailed Student's *t*‐test was used in (b) and one‐way ANOVA followed by Tukey's post hoc test was used in (c) for statistical analysis.

**FIGURE 10:**
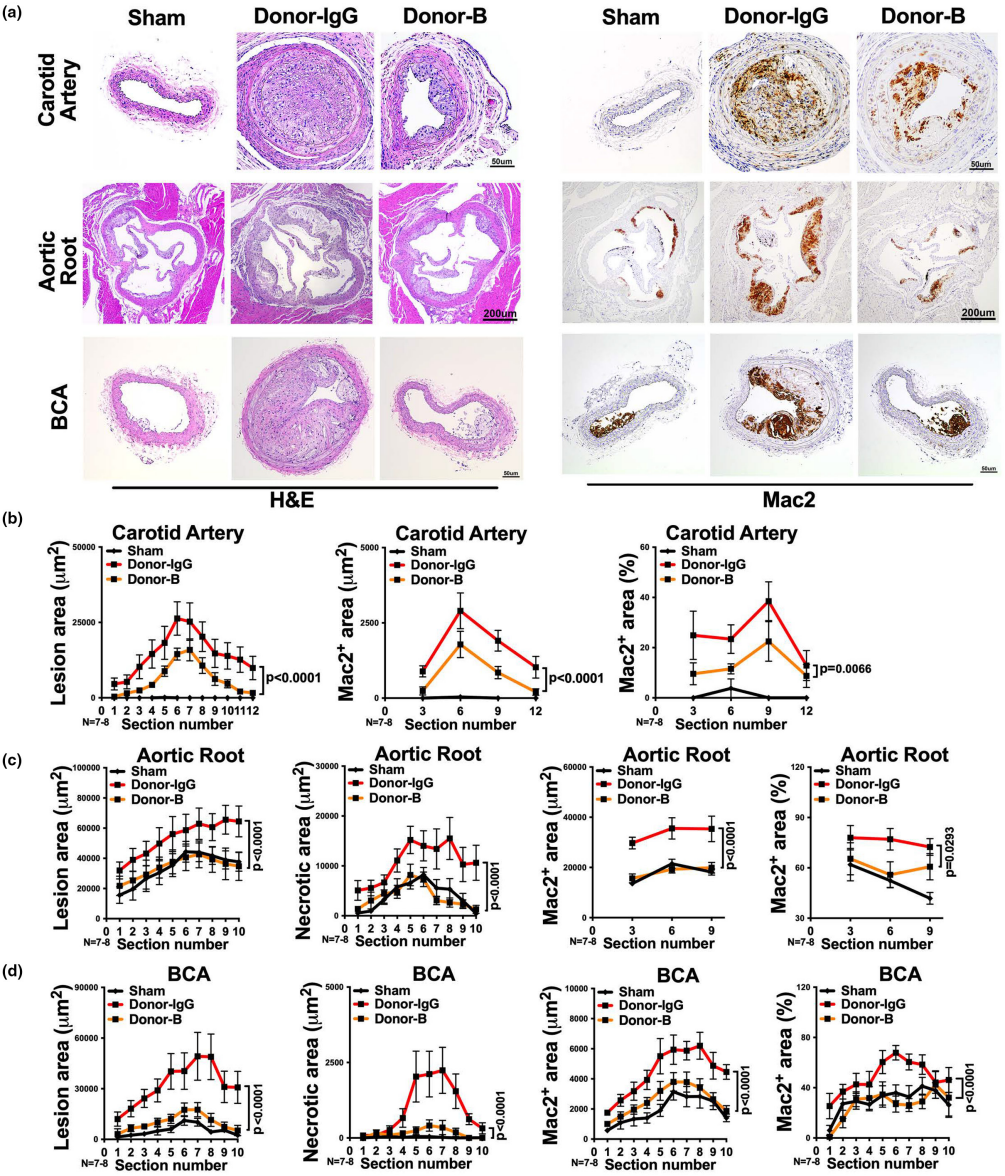
Combined blockade of TNFα, CCL2, and CXCL2 reduces atherogenesis after visceral fat transplantation from aged donors to *Ldlr* ^−/−^ recipients. (a) Representative images of H&E or Mac2 stained right carotid artery, aortic root and BCA sections of *Ldlr* ^−/−^ mice that received sham surgery or visceral fat transplants from IgG control (Donor‐IgG) or TNFα, CCL2, and CXCL2 blockade (Donor‐B) treated aged donor mice. (b–d) The quantifications of lesion area, necrotic area, Mac2 positive area and the percentage of Mac2 positive area in each section of (b) right carotid artery, (c) aortic root and (d) BCA are shown (*n* = 7‐8/group). *Note*: no necrosis was observed in carotid artery. Results are presented as means ± SEM. Two‐way ANOVA followed by Tukey's post hoc test was used for statistical analysis. *p* values indicate the main effect between groups of Donor‐IgG vs. Donor‐B.

We next tested whether combined treatment with anti‐TNFα, anti‐CXCL2, and anti‐CCL2 would reduce the ability of aged fat transplants to enhance atherosclerosis. We therefore administered a combination of neutralizing antibodies including anti‐TNFα, anti‐CXCL2, and anti‐CCL2, or isotype control, to aged donor mice, and then we transplanted the visceral fat from these mice to *Ldlr* ^−/−^ recipients. After transplantation, we continued to treat recipients with IgG control or the “cocktail” blockade till the end of the experiment (Figure 9a). We harvested fat transplants, plasma, and the relevant vascular territories at the end. Recipients that received the combined treatment of neutralizing antibodies exhibited approximately twofold reduction in most of the measured inflammatory mediators secreted by the explanted fat transplants except IL‐1β, including TNFα, IL‐6, CCL2, CCL3, and CXCL2 (Figure 9b). Also, recipients who received the combined blockade exhibited reductions in plasma TNFα, IL‐6, CCL2, and CXCL2, though not IL‐1β or CCL3, compared to recipients treated by IgG control (Figure 9c). Importantly, recipients that received the combined blockade exhibited significantly (*p* < 0.0001) reduced atherosclerotic lesions in carotid artery, aortic root, and BCA, as well as necrotic area and the macrophage burden in all the lesions (Figure 10a–d).

All these results strongly suggest that TNFα, CXCL2, and CCL2 are most importantly and predominantly involved in mediating the aged visceral fat transplant‐enhanced atherogenesis.

## 3 DISCUSSION

Prior to our current study, the role of aging on the adipose tissue to enhance atherosclerosis was unclear. Our study has clarified this issue by first examining the impact of aging on the inflammatory profile of different fat depots and then employing a fat transplant model to determine if aging directly alters the visceral fat depot to promote atherosclerosis. Our study has revealed that visceral fat from aged donors not only directly enhanced atherosclerosis in the vascular territory that is in direct contact to the transplanted fat tissue, but also in distal vascular territories, specifically aortic root and BCA. This indicates that aging of the visceral fat enhances atherosclerosis through secreted factors by both paracrine (i.e., locally) and endocrine (i.e., systemically) mechanisms.

A prior study using heterochronic fat transplantation revealed that aging of visceral fat induces coronary artery vascular dysfunction (Dou et al., 2017). In particular, this study found that transplanting adipose tissue from aged obese mice into the subcutaneous tissue of young recipients led to a TNFα‐dependent systemic effect, which impaired the ability of coronary arteries to respond to endothelium‐dependent coronary dilation (Dou et al., 2017). However, this study did not examine the role of aging in visceral fat and atherogenesis. Another study found that transplanting PVAT from either young or middle‐aged donor mice (i.e., 12 months of age) onto the carotid artery, which was the site of acute injury induced by ferrous chloride, may age‐independently protect from vascular injury (Schütz et al., 2019). However, this study contrasts with an earlier study that found, transplantation of PVAT onto the carotid artery of *Ldlr* ^−/−^ mice increased acute wire injury in a CCL2 dependent manner (Manka et al., 2014). Another study that transplanted visceral fat from young donor mice directly onto the carotid artery of young *ApoE* ^−/−^ mice, a murine model of atherosclerosis (Daugherty et al., 2017), found that the fat transplant enhanced atherosclerosis within the carotid artery, a phenotype associated with increased serum CCL2 levels (Ohman et al., 2011). However, this study did not examine the effect of aging on visceral fat and subsequent atherosclerosis.

Our study substantially extends the above studies by demonstrating that aging increases the inflammatory profile of visceral fat to enhance atherosclerosis after visceral fat transplantation onto the right carotid artery of young *Ldlr* ^−/−^ mice. We found that recipients of aged fat transplants exhibited increased plasma levels of TNFα and CXCL2 (Figure 8b). Additionally, we found that inhibiting CCL2 in the culture supernatant of aged fat transplants impeded the ability of the culture supernatant to enhance monocyte chemotaxis. Based on these results, we examined if giving *Ldlr* ^−/−^ recipients antibodies that block TNFα, CXCL2 and CCL2 would reduce the ability of aged visceral fat transplants to enhance atherosclerosis. We found that the combined inhibition of TNFα, CXCL2 and CCL2 in recipients with aged fat transplants substantially reduced the degree of atherosclerosis at the carotid artery, aortic root and BCA. Young *Ldlr* ^−/−^ mice fed on a western diet do not typically exhibit atherosclerosis within the carotid artery (Figures 2b, 6a,b and 10a,b). Since the combined blockade reduced atherosclerosis within the carotid artery, the site in which the fat transplant was located (Figure 10a,b), our results suggest that the combined blockade of TNFα, CXCL2 and CCL2 reduced the ability of aged visceral fat to enhance atherosclerosis, at least locally. However, our study does not exclude the possibility that other inflammatory mediators, such as IL‐6 and IL‐1β, that are secreted excessively by the visceral fat with aging may contribute to atherogenesis. Furthermore, our study has not determined which individual inflammatory mediator secreted by aged visceral fat is most critical for atherosclerosis via a paracrine mechanism (i.e., enhancing atherosclerosis at the site of transplantation at carotid artery) or via an endocrine mechanism (i.e., enhancing atherosclerosis at distal sites such as aortic root and BCA). Deciphering these potential complex mechanisms in vivo will clearly require future investigations.

Typically, macrophages within atherosclerotic lesions are derived from circulating monocytes that migrate to the vessel wall and differentiate into macrophages after transmigration into the vessel wall (Moore et al., 2013; Tacke et al., 2007), although embryonically derived tissue resident macrophages within the vessel wall may also contribute to atherosclerosis (Robbins et al., 2013). Our study provides evidence that aged visceral fat secrets increased levels of monocyte‐attracting mediators and induces a higher degree of monocyte chemoattraction than young visceral fat (Figures 7a and 8a).

A prior study has shown that deleting the monocyte chemokine CCL2, along with the monocyte chemokine receptors CX3CR1 and CCR5 (via crossing mice with genetic deficiency of each receptor to the *ApoE* ^−/−^ background), abrogates atherogenesis (CombadièRe et al., 2008). Expression of these chemokine receptors and CCR2, the receptor for CCL2, indicates an activated or atherogenic phenotype in monocytes (Gautier et al., 2009; Tacke et al., 2007). Our study shows that culture supernatants from aged fat transplants induces upregulation of the “atherogenic” chemokine receptors CCR2, CCR5, and CX3CR1 (Figure 7c). Based on our results and those in the literature (Gautier et al., 2009; Tacke et al., 2007), we speculate that excessive secretion of inflammatory mediators by aged visceral fat transplants induces an atherogenic phenotype in circulating monocytes that increases their homing into the vessel wall of hyperlipidemic hosts to enhance atherosclerosis.

Our study has provided evidence that macrophages within the aged visceral fat depot are the major contributors to the secretion of inflammatory mediators. Depleting macrophages from visceral fat of aged mice reduced the secretion of all measured inflammatory mediators by 2–10‐fold (Figure 4c). When we transplanted clodronate treated aged visceral to recipients, the ability of aged fat transplants to enhance atherosclerosis was reduced at least twofold (Figure 6). We have no specific data to support the notion that adipose tissue macrophages within fat transplants are directly migrating to the atherosclerotic lesions. Instead, based on our data, it's likely that macrophages within the aged visceral fat are producing inflammatory mediators that ultimately lead to the upregulation of the atherogenic phenotype on circulating monocytes, which subsequently infiltrate into atherosclerotic lesions and enhance atherogenesis. Finally, a prior study found that clodronate treatment of young donor mice failed to reduce the ability of visceral fat to enhance atherosclerosis after transplantation to young *Ldlr* ^−/−^ mice (Bijnen et al., 2019). However, this study transplanted the visceral fat into the peritoneal cavity of the recipients, in contrast to the carotid artery, which was the site employed in our study, and this study did not examine the effect of aging on visceral fat to enhance atherosclerosis. These important differences may explain the different results between our study and the study by Bijnen et al., 2019.

Our study provides evidence that aged visceral fat exhibited increased expression of the senescent marker β‐galactosidase (Debacq‐Chainiaux et al., 2009), compared to visceral fat from young mice. Senescence is accompanied by secretion of inflammatory mediators, a phenomenon known as SASP (Tchkonia et al., 2013). Our study found that aging increases the ability of the visceral fat depot to secrete a broad range of inflammatory mediators compatible with the SASP phenotype. These results are consistent with the prior notion that aging increases senescence within adipose tissue (Liu et al., 2020; Ou et al., 2022; Tchkonia et al., 2010; Xu et al., 2015). Given this, we examined if removal of senescence cells, via senolytics, would reduce the secretion of inflammatory mediators from aged visceral fat. We found that senolytics reduced the secretion of all measured inflammatory mediators (Figure S4). As the ability of aged visceral fat to secrete inflammatory mediators was not completely abrogated by senolytics, suggesting that other biological hallmarks of aging may be operational in the visceral fat with aging (López‐Otín et al., 2013). For instance, mitochondrial dysfunction may contribute to the increased inflammation of aged visceral fat (Macêdo et al., 2021; Vernochet & Kahn, 2012; Woo et al., 2019). Senolytics have been shown in murine models to reduce atherosclerosis (Roos et al., 2016), but whether they act via effects on visceral fat is not clear. Senolytics, or strategies to improve mitochondrial dysfunction such as antioxidants, may mitigate some of the proatherogenic effects of aging on visceral fat, but will require future investigation to answer.

In conclusion, our study has revealed that aging increases the inflammatory profile of visceral fat to directly promote atherosclerosis. Our study indicates that with aging, macrophages within the visceral fat are major contributors to the enhanced inflammatory profile of visceral fat, potentially by secreting TNFα, CXCL2, and CCL2, to promote atherogenesis (graphically depicted in the graphical abstract). If the findings of our study are translated to humans, potential future therapeutics aimed at reducing atherosclerosis in the expanding older population should consider targeting the adipose tissue.

## 4 MATERIALS AND METHODS

### 4.1 Animal

All mice used in this study were on C57BL/6 background, except for the outbred UM‐HET3 mice (see below), and all of them were male unless specifically indicated. We employed 3–4 months old wild‐type (WT) young mice and 19–21 months old WT aged mice in this study. The mice were obtained from the National Institute of Aging rodent colony at Charles River breeding laboratories. Mice were monitored for around 1 month after delivery before they were used for experiments. 3–5 months old low density lipoprotein receptor knockout (*Ldlr* ^−/−^) mice were used as recipients in experiments with fat transplant surgery. The *Ldlr* ^−/−^ mice were originally purchased from Jackson Laboratory (stock #002207) and then bred and housed in animal facility at the North Campus Research Complex at the University of Michigan. Male UM‐HET3 mice, a four‐way crossed outbred mouse strain used by the National Institute on Aging Interventions Testing Program (Miller et al., 2007), were generously provided by Dr. Richard Miller at the University of Michigan. These mice were aged at the Glenn Center at the University of Michigan. Unless otherwise indicated, the mice were maintained on low‐fat standard laboratory chow diet (5L0D, LabDiet) and water ad libitum. The number of mice for each experiment are shown in the figure legends.

### 4.2 Fat transplantation

A 100 mg of visceral fat (we used gonadal white adipose tissue, GWAT) was removed from donor mice and implanted adjacent to the right common carotid artery (RCCA) of young *Ldlr* ^−/−^ mice that were served as recipients. Recipients were anesthetized with 2% isoflurane during the surgery. Carprofen (5 mg/kg) was injected subcutaneously before the surgery and then 24 and 48 h later. For sham controls, identical procedures were performed without fat implantation. Mice were randomly assigned to fat transplant intervention or sham groups. Recipients were fed a laboratory chow diet (5L0D, LabDiet) before and 1 week after the surgery (to facilitate healing) followed by a Western diet (42% calories from fat, #88137, Teklad) for 10 weeks to increase cholesterol levels and promote atherosclerosis.

For experiments that include young and aged mice, young or aged mice were used as donors. For experiments that include macrophage depletion, aged mice that were treated by control liposome or liposome‐encapsulated clodronate (SKU# CLD‐8901, Encapsula NanoSciences) were used as donors. Liposomes containing 1 mg/10 g of clodronate or an equivalent volume of control liposomes were given each mouse intraperitoneally. Macrophage depletion in GWAT was confirmed after 2 days. For experiments that include the blockade, aged mice treated by IgG control or the combination of TNFα, CCL2 and CXCL2 blockade were used as donors. Mouse antibodies against TNFα (Cat# BE0058) and CCL2 (Cat# BE0185) were purchased from Bio X Cell, and anti‐CXCL2 (Cat# MAB452) was from R&D Systems. Anti‐TNFα (500 μg/mouse) and anti‐CCL2 (200 μg/mouse) were injected intraperitoneally and anti‐CXCL2 (100 μg/mouse) was injected intravenously into donors 2 days prior to transplantation, and into recipients after transplantation twice per week till the end of the experiment. Mice treated with IgG served as controls.

### 4.3 Histopathology

Histology services were performed by the In Vivo Animal Core histology laboratory within the Unit for Laboratory Animal Medicine at the University of Michigan. Briefly, formalin‐fixed tissues were processed through graded alcohol and cleared with xylene followed by infiltration with molten paraffin. Tissues were then sectioned at 4 μm thickness. Sectioning paradigms for the vasculature: carotid artery, spanning approximately 1 mm, 100 micron step levels and a total of 12 sections were collected; aortic root, spanning approximately 250 microns, 24 micron step levels, and a total of 10 sections were collected beginning at the aortic valve leaflets; BCA, beginning at the proximal root of the BCA, 100 micron step levels and a total of 10 sections were collected until the right common carotid artery / right subclavian artery junction was reached. To determine the atherosclerotic lesion size and the acellular lesion (necrotic core) area (Daugherty et al., 2017; Fernandez‐Hernando et al., 2007; Paigen et al., 1987), sections were subjected to hematoxylin and eosin (H&E) staining and then traced and measured using ImageJ. A total of 12 H&E stained sections from carotid artery, 10 H&E stained sections from aortic root and 10 H&E stained sections from BCA were quantified per mouse. Immunohistochemical staining was performed to detect the macrophage markers of Mac2 (Santa Cruz, Cat# sc‐81728, 1:100 dilution) and F4/80 (Bio‐Rad ABD Serotec, Cat# MCA497, 1:400 dilution), and nucleus was counterstained with hematoxylin. For analysis of macrophages in aortic root, section #3, #6, and #9 were chosen. For macrophages staining in carotid artery in Figures 6 and 10, section #3, #6, #9, and #12 were chosen. The percentage of Mac2 positive area in indicated sections was calculated with the following equation:

$${\text{Mac2}}^{+}\;\text{area}\;\left(\%\right)={\text{Mac2}}^{+}\;\text{area}/\left(\text{lesion area}–\text{necrotic area}\right)\times 100\%.$$

### 4.4 Flow cytometry

Adipose tissue fractionation and flow cytometric analysis were performed to identify adipose tissue macrophage (Singer et al., 2014). Briefly, whole adipose tissue was minced and digested with type II collagenase (1 mg/mL, Sigma, #C6885) for 15 to 30 min at 37°C on a rocker. Samples were filtered and centrifuged, and red blood cell lysis was conducted on SVF. SVF single cell suspensions were then incubated with the antibodies. The following antibodies were used: CD45 eFluor450 (Clone 30‐F11, Invitrogen), CD64 PE (Clone X54‐5/7.1, BD Pharmingen), CD11c APC eFluor780 (Clone N418, Invitrogen), CD45 FITC (Clone 30‐F11, Biolegend), F4/80 PE (Clone BM8, Biolegend), and CD11b BV421 (Clone M1/70, Biolegend). Flow cytometry was performed using a BD LSRFortess flow cytometer and analyzed with FlowJo V10.7 software. Macrophages were defined as CD45^+^CD64^+^ or CD45^+^CD11b^+^F4/80^+^, and infiltrated macrophages were defined as CD45^+^CD64^+^CD11c^+^(Singer et al., 2015).

### 4.5 Multiplex cytokine analysis

V‐PLEX assays from MSD (Meso Scale Discovery) multi‐spot assay system were used to quantify cytokines in the tissue culture medium of transplanted visceral fat tissues (transplanted GWAT, tGWAT) and the plasma of fat transplant recipients. Customized panels were used according to the manufacturer's instructions. Panel 1 (Cat# K15048D) includes TNFα, IL‐6, and IL‐1β, and panel 2 (Cat# K15245D) includes CCL2, CCL3, and CXCL2.

### 4.6 Chemotaxis assay

Monocytes were isolated from the bone marrow of young mice by immunomagnetic negative selection (StemCell, Cat#19861). Boyden chambers, specifically Cell Biolabs CytoSelect™ 96‐well Cell Migration Assay Kit (5 μm pore size, Cat# CBA‐105‐5) was used to investigate the chemotaxis of monocytes toward the culture media of young or aged fat transplants. The assay was performed according to the manufacturer's instructions. Briefly, 100 μl of 0.5–1.0 × 10^5^ cells/ml bone marrow derived monocytes were loaded in the membrane chamber in serum free media, and 150 μl of tissue culture media from young or aged fat transplants was added to the wells of the feeder tray as chemoattractant. By the end of the incubation, the migrated cells were lysed in 4× Lysis Buffer/CyQuant® GR dye solution, and fluorescence was read with a fluorescence plate reader at 480 nm/520 nm. Anti‐CCL2 (100 μg/ml, Bio X Cell, Cat# BE0185), anti‐TNFα (25 μg/ml, Bio X Cell, Cat# BE0058), and/or anti‐CXCL2 (50 μg/ml, R&D Systems, Cat# MAB452) or isotype control were used to block the corresponding cytokine(s) in the culture media. Serum‐free medium was used as negative control for the culture media.

### 4.7 Quantitative RT‐PCR

Migrated monocytes were collected from the tissue culture media in 24 well Transwell plates (5 μm pore size, Costar, Cat# 3421). Relative mRNA expression was determined using quantitative reverse‐transcription polymerase chain reaction (qRT‐PCR). Total RNA was extracted using TRIzol reagent and RNA was reverse transcribed to cDNA with a High‐Capacity cDNA Reverse Transcription Kit (Applied Biosystems). Quantitative polymerase chain reaction was performed using a 7900HT fast real‐time PCR system (Applied Biosystems) and relative mRNA expression was analyzed using the comparative method and normalized to the internal control, L32. Primer sequences for qRT‐PCR reactions are shown in Table S1.

### 4.8 Statistical analysis

Results are presented as mean ± SEM. For statistical analysis, unpaired two‐tailed Student's *t*‐test, one‐way ANOVA and two‐way ANOVA followed by Tukey's post hoc test were used. Figures that used two‐way ANOVA, *p* values indicate the main effect between the indicated treatments. No interaction terms between treatment and location (section #) was found significant. All statistical analysis was performed in GraphPad Prism (GraphPad Software, Inc). *p* < 0.05 was considered significant.

### 4.9 Study approval

Animal protocols were approved by the University of Michigan Animal Care and Use Committee. All animal procedures were performed in accordance with the Guide for Care and Use of Laboratory Animals and conformed to the NIH guidelines.

## AUTHOR CONTRIBUTIONS

JS: designing research studies, conducting experiments, acquiring and analyzing data, writing the manuscript. DF: conducting experiments, particularly the fat transplant surgery. PA: acquiring and analyzing data. SM: conducting experiments and editing manuscript. MV and KS: assistance in acquiring flow cytometric data, editing manuscript. MB, JC and DT: conducting experiments, analyzing data and editing manuscript. MZ: analyzing data, writing the manuscript. MS: analyzing data, writing the manuscript. DRG: designing research studies, analyzing data, writing the manuscript, procuring funding.

## CONFLICT OF INTEREST

The authors declare no conflict of interest exist.

## Supporting information


Appendix S1

## Data Availability

Data is available on request from the authors. The data that support the findings of this study are available from the corresponding author upon reasonable request.
