# Supplementary material for: Age‐associated adipose tissue inflammation promotes monocyte chemotaxis and enhances atherosclerosis
Source: Aging Cell. 2023 Jan 23;22(2):e13783. doi: 10.1111/acel.13783 (PMC9924943; doi:10.1111/acel.13783)
Supplement: Supplementary file 1 — Appendix S1 [file ACEL-22-e13783-s001.docx]

Age-associated adipose tissue inflammation promotes monocyte chemotaxis and enhances atherosclerosis

**Supplemental Materials**

**Supplemental Figure 1. Aging increases visceral fat inflammation is not restricted to specific genetic background or gender. (A**) Visceral fat (GWAT) from young (Y) and aged (A) male UM-HET3 mice were stained for Mac2: image shown on left, quantification shown on right. **(B)** Visceral fat (GWAT) from young and aged female mice were stained for Mac2: image shown on left, quantification shown on right. **(C-D)** Protein levels of TNFα, IL-6, IL-1β, CCL2, CCL3 and CXCL2 in the tissue culture medium (CM) of **(C)** visceral adipose tissue (GWAT) from female mice (n=6-7/group) and **(D)** subcutaneous adipose tissue (IWAT) from male mice (n=12-14/group) were determined by multiplex assay. **(E)** Indicated fat tissues from young and aged male or female mice were cultured *ex vivo* and the level of leptin in the conditioned media (CM) was determined by ELISA. Results are presented as means ± SEM. Unpaired two-tailed Student’s t-test was used for statistical analysis.

**Supplemental Figure 2. Fat transplantation does not affect the metabolism of recipients.** Fasting serum was collected at indicated time points from *Ldlr^-/-^* recipients that received sham surgery or visceral fat (GWAT) transplants from young (Donor-Y) or aged (Donor-A) mice. Fasting cholesterol, triglyceride, insulin levels and the body weight of recipients were measured accordingly (n=6-8/group). Results are presented as means ± SEM. 2-way ANOVA was used for statistical analysis.

**Supplemental Figure 3. Aged fat transplants increase the percentage of Mac2 staining in aortic root and BCA in recipients.** The percentage of Mac2 positive area in indicated sections of right carotid artery, aortic root and BCA of *Ldlr^-/-^* mice that received sham surgery or visceral fat transplants from young (Donor-Y) or aged (Donor-A) mice are shown (n=7-10/groups). Results are presented as means ± SEM. 2-way ANOVA followed by Tukey’s post hoc test was used for statistical analysis. P values indicate the main effect between groups of Donor-Y and Donor-A.


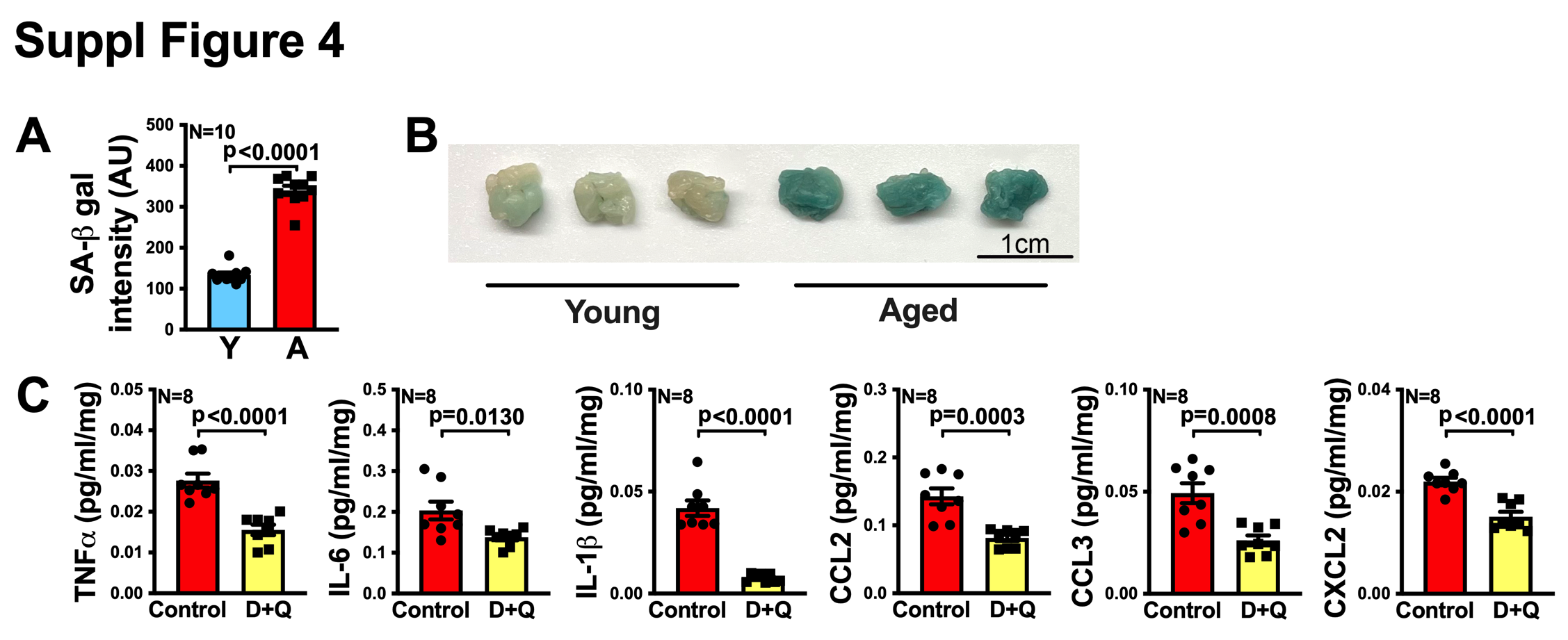


**Supplemental Figure 4. Senescence contributes to the inflammatory profile of aged fat. (A and B)** Senescence-associated β-galactosidase (SA-β-gal) staining in visceral fat (GWAT) of young (Y) and aged (A) mice. **(A)** Quantification (n=10/group) and **(B)** representative pictures of SA-β-gal staining. AU, arbitrary unit. **(C)** Protein levels of TNFα, IL-6, IL-1β, CCL2, CCL3 and CXCL2 in tissue lysates of visceral fat (GWAT) from aged mice were determined by multiplex assay (n=8/group). Those aged mice were treated by either vehicle control or senolytic drugs dasatinib and quercetin (D+Q) through oral gavage.

**Supplemental Materials and Methods**

**ELISA**

Tissue culture medium and plasma were collected from indicated tissues or mice. ELISA kit of Leptin (Invitrogen, Cat# KMC2281) was used according to the manufacturer’s instructions.

**Cholesterol, Triglyceride and Insulin measurements**

Fasting serum was collected at indicated time points by tail bleeding. Cholesterol levels were determined by colorimetric assay (Cat# STA-384, Cell Biolabs). Serum triglyceride levels were measured with a colorimetric assay too (Cat# STA-396, Cell Biolabs) following manufacturer’s instructions. Fasting insulin levels were determined with an ultrasensitive insulin ELISA kit (Cat# 90080, Crystal Chem).

**SA–β-Gal Staining**

Senescence associated β-galactosidase (SA-β-gal) activity in visceral fat (GWAT) from young and aged mice was determined by a SA–β-gal staining kit (Cat# 9860S, Cell Signaling Technology). The quantification was performed using Image J and SA-β-gal values are reported as arbitrary units (AU).

**Dasatinib and Quercetin (D+Q) Treatment**

Dasatinib (Millipore Sigma, SML2589) and Quercetin (Millipore Sigma, Q4951) were dissolved in a solution of 10% ethanol (Millipore Sigma, C7023), 30% polyethylene glycol 400 (Millipore Sigma, 8074850050) and 60% Phosal 50 PG (Fisher Scientific, NC0130871). Aged mice were given either D+Q (Dasatinib 5mg/kg, Quercetin 50mg/kg) treatment or vehicle control by oral gavage for 3 consecutive days every 2 weeks for a total of 6 treatments over the span of a month.

**Supplemental Table. 1**

| **Gene** | **Forward** | **Reverse** |
| --- | --- | --- |
| CCL2 | TCACCTGCTGCTACTCATTCACCA | TACAGCTTCTTTGGGACACCTGCT |
| CCL3 | TTCTCTGTACCATGACACTCTGC | CGTGGAATCTTCCGGCTGTAG |
| CCR2 | TGTGATTGACAAGCACTTAGACC | TGGAGAGATACCTTCGGAACTT |
| CCR5 | TTTTCAAGGGTCAGTTCCGAC | GGAAGACCATCATGTTACCCAC |
| CXCL2 | GCGCTGTCAATGCCTGAAGTC | CGTCACACTCAAGCTCTGGAT |
| CX3CR1 | GAGTATGACGATTCTGCTGAGG | CAGACCGAACGTGAAGACGAG |
| IL-1β | AAGAGCTTCAGGCAGGCAGTATCA | TGCAGCTGTCTAATGGGAACGTCA |
| IL-6 | GAGGATACCACTCCCAACAGACC | AAGTGCATCATCGTTGTTCATACA |
| L32 | TTAAGCGAAACTGGCGGAAAC | TTGTTGCTCCCATAACCGATG |
| MMP2 | CAAGTTCCCCGGCGATGTC | TTCTGGTCAAGGTCACCTGTC |
| MMP12 | AATGCTGCAGCCCCAAGGAAT | CTGGGCAACTGGACAACTCAACTC |
| TGFβ1 | CTCCCGTGGCTTCTAGTGC | GCCTTAGTTTGGACAGGATCTG |
| TNFα | CCCTCACACTCAGATCATCTTCT | GCTACGACGTGGGCTACAG |
| VEGFc | CTGTCCTGGTATTGAGGGTGG | GAGGTCAAGGCTTTTGAAGGC |

**Supplemental Table 1. Sequences of primers used in this study.** The primers were synthesized by Integrated DNA Technologies. L32, 60S ribosomal protein L32, was used as internal control.
